# An optical tracker based robot registration and servoing method for ultrasound guided percutaneous renal access

**DOI:** 10.1186/1475-925X-12-47

**Published:** 2013-05-24

**Authors:** Dongwen Zhang, Zhicheng Li, Ken Chen, Jing Xiong, Xuping Zhang, Lei Wang

**Affiliations:** 1 Shenzhen Key Laboratory for Lowcost Healthcare, Key Lab for Health Informatics, Shenzhen Institutes of Advanced Technology, Chinese Academy of Sciences, Xueyuan Avenue 1068, Shenzhen 518055, China; 2 University of Chinese Academy of Sciences, No.19A Yuquan Road, Beijing 100049, China

## Abstract

**Background:**

Robot-assisted needle steering facilitates the percutaneous renal access (PRA) for their accuracy and consistency over manual operation. However, inaccurate image-robot correspondence and uncertainties in robot parameters make the needle track deviate from the intrarenal target. This paper aims to simplify the image-tracker-robot registration procedure and improves the accuracy of needle alignment for robot assisted ultrasound-guided PRA.

**Methods:**

First, a semi-automatic rigid registration is used for the alignment of the preoperative MR volume and the intraoperative orthogonal US slices. Passive markers are mounted both on US probe and robot end-effector, the planned puncture path is transferred from the MR volume frame into optical tracker frame. Tracker-robot correspondence and robot calibration are performed iteratively using a simplified scheme, both position and orientation information are incorporated to estimate the transformation matrix, only several key structural robot parameters and joint zero-positions are calibrated for simplicity in solving the inverse kinematic. Furthermore, an optical tracker feedback control is designed for compensating inaccuracies in robot parameters and tracker-robot correspondence, and improving the accuracy of needle alignment. The intervention procedure was implemented by a telemanipulated 5R1P robot, two experiments were conducted to validate the efficiency of robot-tracker registration method and the optical tracker feedback control, robot assisted needle insertion experiment was conducted on kidney phantom to evaluate the system performance.

**Results:**

The relative positioning accuracy of needle alignment is 0.24 ± 0.08 mm, the directional accuracy is 6.78 ± 1.65 × 10^-4^rad; the needle-target distance of needle insertion is 2.15 ± 0. 17 mm. The optical tracker feedback control method performs stable against wide range of angular disturbance over (0 ~ 0.4) radians, and the length disturbance over (0 ~ 100) mm.

**Conclusions:**

The proposed optical tracker based robot registration and servoing method is capable of accurate three dimension needle operation for PRA procedure with improved precision and shortened time.

## Background

Robot-assisted needle insertion facilitates many minimally invasive percutaneous procedures such as biopsy, electrolytic ablation and renal intervention, where a similar establishment of reliable and consistent access track from skin to the inside anatomical feature is required. In percutaneous renal intervention, it is important to locate the needle tip as well as the track of the needle shaft under intra-operative guidance of x-ray or ultrasound images [1-3]. Accurate steering and placement of needle is challenging due to uncertainties in image-robot correspondence, which makes the needle track deviate from the target.

Numbers of robotic systems have been proposed for eliminating radiation exposure and simultaneously increasing accuracy in radiologic interventions. Bzostek et al. [1] used a stereo-pair of two x-ray views registered to a common fiducial system with an active robot to assist needle placement. Yu Zhou et al. introduced a CT-guided robotic needle biopsy technique for lung nodules. Based on the nodule respiratory motion model, needle placement is planned to follow an optimal timing and path, and is triggered based on the respiratory phase tracking [4]. The PAKY-RCM incorporates a passive robotic arm and a friction transmission with axial loading system, which allows a urologist to remotely align the needle along a selected trajectory path under fluoroscopic guidance using the superimposed registration principle [2]. These methods all require time consuming pre-operative registration procedures between robot, imaging system and the patient's anatomy. Patriciu et al. uses the laser markers readily available on any CT scanner for robot registration in computer tomography imaging systems. This approach does not require additional hardware, laser alignment being performed on the instrument used in the clinical application [5]. An automatic image-guided control based on visual servoing and principles of projective geometry is presented for automatic and uncalibrated needle placement under CT-fluoroscopy. The approach demonstrated good targeting accuracy by using the procedure needle as a marker, without additional registration hardware [6].

Robotic percutaneous interventions guided by ultrasound are developed in recent decades for that ultrasound (US) is radiation-free, real-time and easy-to-use [7]. J Hong et al. proposed an ultrasound-driven needle insertion robot for percutaneous cholecystostomy, which is capable of modifying the needle path by real-time motion compensation through visual servo control before needle insertion [8]. Robot assisted and ultrasound guided ablative therapy and biopsy operation are also studied [9,10], an optical/electromagnetic marker mounted on ultrasound probe are used to measure the transducer’s position and orientation, once the puncture path is defined, the robotic arm moved automatically to the planned insertion path. Ultrasound image-based visual servoing techniques have not been used in percutaneous interventions for that the abdominal US is often related to limited anatomy identification and targeting abilities, providing only two-dimensional(2D) anatomical information with poor quality [3,11,12].

In our previous works, an augmenting intraoperative ultrasound with preoperative magnetic resonance planning models for PRA was proposed and evaluated by urologists on a kidney phantom. With careful setup it can be efficient for overcoming the limitation of current US-guided PRA [13,14]. In this paper, a telemanipulated 5R1P robot is employed for needle operation. We present an optical tracker based robot registration and servoing method for ultrasound-guided PRA, optical tracker serves as intermediate coupling tool for image-robot registration and error feedback control for needle alignment. The rest of the paper is organized as follows, introduction of experiment setup and navigation systems, image-robot registration and robot control scheme are illustrated in Sec. II. The last two sections describe the experiment and discussions.

## Methods

### Procedures of robot assisted percutaneous renal intervention

The robot assisted percutaneous renal intervention surgery workflow consists of preoperative surgical planning, intraoperative surgical navigation and semi-autonomous telemanipulated needle operation. First, the patient is scanned by magnetic resonance (MR), kidney, vessels and tumor are then segmented from the MR volume as 3D model, such that a surgeon can make a optimal surgical plan preoperatively. During the surgery, a semi-automatic rigid registration is performed for the alignment of the US slices and the MR volume, the preoperative planning can be transferred onto the patient in situ. With an image-guidance interface, the surgeon guide the robot to the insertion point, needle alignment and interventional puncture can be performed autonomously in accordance with the surgical planning. Verification that the needle has successfully gained access to the collecting system will be provided by the return of urine through the trocar needle. The needle will be detached from the robot, and subsequent surgical procedure continues.

### Experiment setup

**Figure 1:**
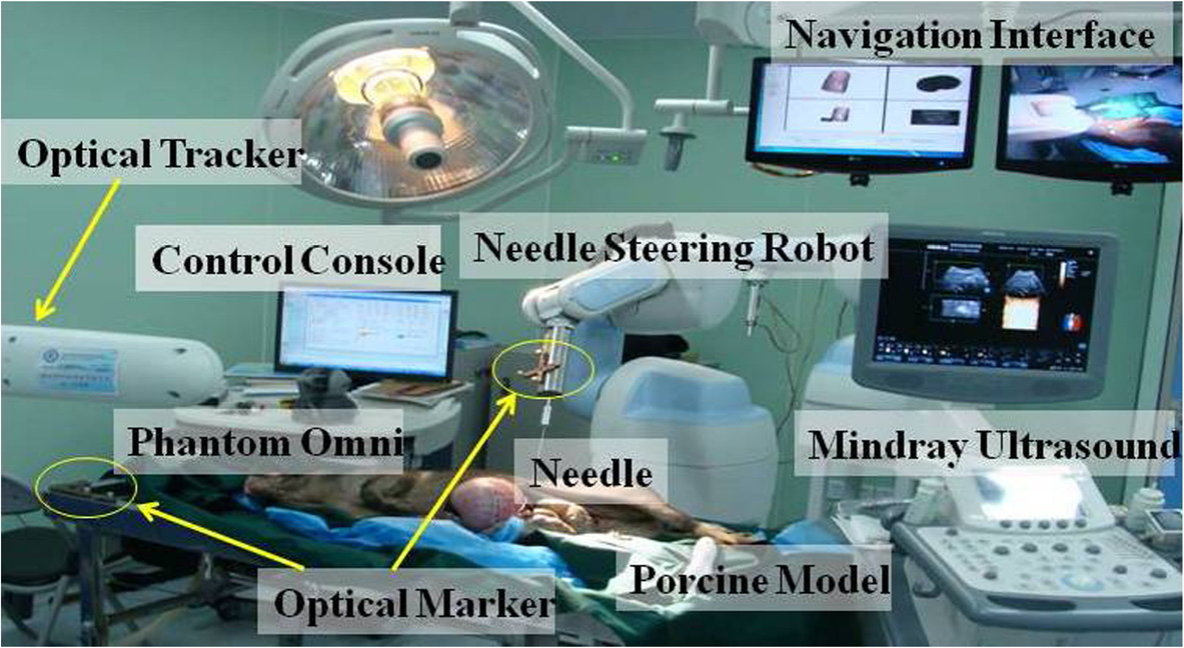
Block diagram of the master–slave experimental set-up for needle intervention.

The prototype system for needle insertion has been set up in our integrated operating room (see Figure 1). The master was the PHANToM OMNI haptic device (SensAble Technologies Inc., USA) ,which provided force position measurements at its end point and feedback in three DOFs. A 5R1P industrial robot (REBo-V-6R-650, Shenzhen Reinovo Technology Co. Ltd., China) was employed for needle operation, five rotational joints uniquely determine the needle orientation and position, the last linear DC-servomotor (Quickshaft LM1247, Faulhaber Group, Germany) served for needle insertion with positional accuracy 180 μm and maximum 3N force. The last two joint axes of the wrist and the translational axis are intersecting in a single point. Needle orientation and puncture are independently activated by the corresponding joints and safe button of the haptic device. An 18-gauge trocar needle (090020-ET, Cook Urological Inc., USA) with a triangular diamond tip was attached to the end-effector by a detachable unit, which was equipped with a force sensor (AL311-BL, Honeywell Inc., USA), the force value were collected by data acquisition card (DAQ 6229-USB card, NI Inc., USA). A 3D ultrasound system (DC-7, Mindray Medical Ltd.,China) was used as an intraoperative navigator. In order to track the 6D positions of needle and ultrasound frame, passive optical markers were mounted to ultrasonic probe and needle holder, the receiver was optical tracking systems with positioning accuracy RMS error 0.35 mm (Polaris Spectra, Northern Digital Inc. (NDI), Canada). All experiments were conducted on the silicon phantom from Computerized Imaging Reference Systems (CIRS), no ethical concern is involved.

### Registration of image-robot-tracker

Registration of the robot to the image space provides us with the essential relationship between the needle location and the targets in image coordinate. Indeed, inaccurate robot-image calibration has a direct impact on the accuracy of the needle steering.

(i). Image-Tracker registration

**Figure 2:**
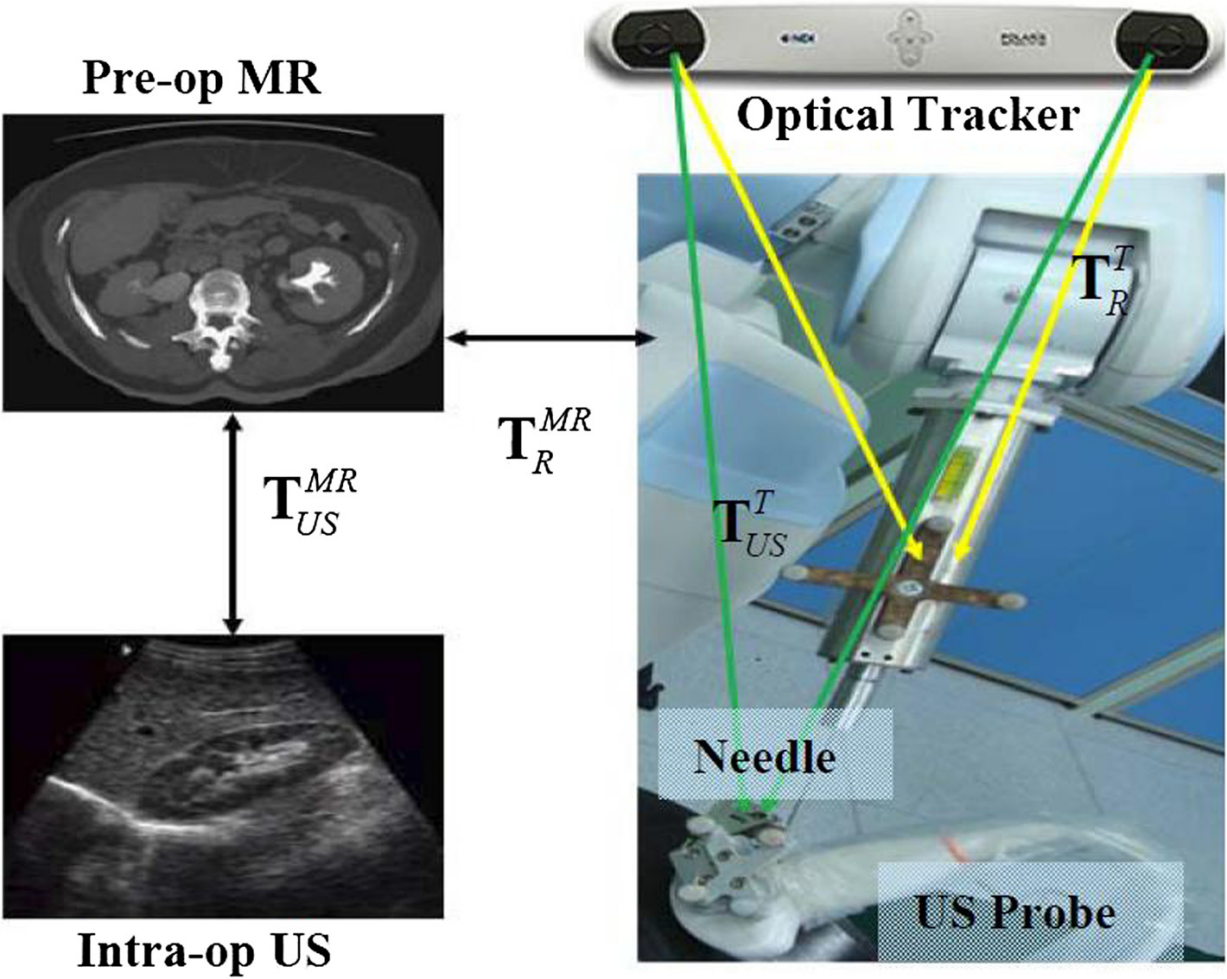
Image-Tracker-Robot registration, the optical tracker acts as an intermediate coupling tool.

At preoperative surgical planning stage, we applied the semi-autonomous algorithm from [15] to segment kidney parenchyma and vascular structures from magnetic resonance images. A 3D plan can be then defined in MR volume coordinate frame **F**_*MR*_ by the surgeon. During the surgery, the tracker reads the positions of maker fixed on the robot end-effector and the US probe, while the preoperative data and the surgical plan are registered to the calibrated intraoperative US images. First, two pairs of orthogonal ultrasound images were collected near the 11th intercostals space at the maximum exhalation positions, all images should contain clearly visible kidney contours. Next, the iterate closet point (ICP) algorithm was performed for the alignment of the US slices and the MR volume, using kidney surface and large vessel surface as registration features [16]. Based on ultrasound-MR volume transformation $T_{\mathrm{US}}^{\mathrm{MR}}$ and tracker-ultrasound transformation $T_{\mathrm{US}}^{T}$, the planned puncture path can be transferred from the preoperative MR volume frame **F**_*MR*_ into intraoperative tracker frame **F**_*Tracker*_. Once robot-tracker registration $T_{R}^{T}$ is done, the surgical plan can be transferred from preoperative MR volume frame **F**_*MR*_ to robot frame **F**_*Robot*_. At the maximum exhalation, the needle is rapidly inserted into the intrarenal target under navigated guidance. The image-robot-tracker registration is shown in Figure 2.

(1) $$T_{R}^{\mathrm{MR}}=T_{\mathrm{US}}^{\mathrm{MR}}{\left(T_{\mathrm{US}}^{T}\right)}^{-1}T_{R}^{T}$$

(ii). Robot-Tracker correspondence

In this section, we propose a simplified registration method for both robot-tracker correspondence and robot calibration.

**Figure 3:**
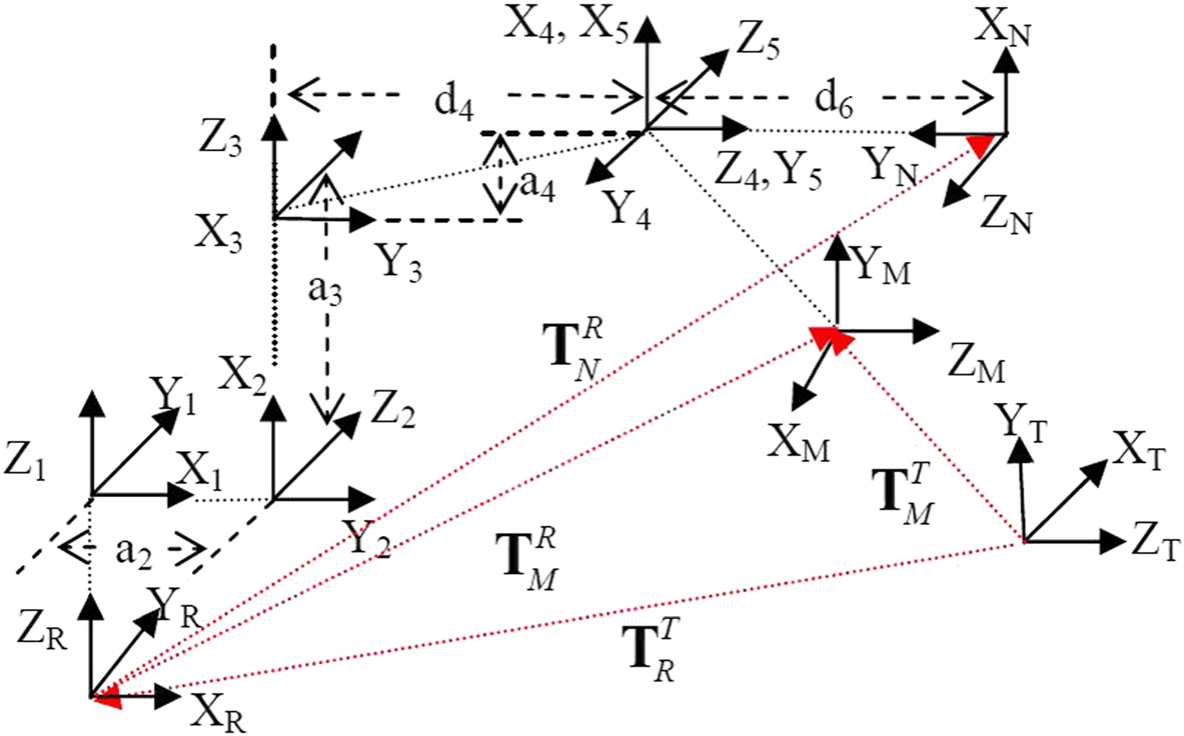
Coordinate systems of the 5R1P needle operation robot.

The coordinate systems of the 5R1P needle operation robot is depicted in Figure 3, frame **F**_*Tracker*_ = (**x**_*T*_,**y**_*T*_,**z**_*T*_) is attached to the optical tracker base, there are total 8 coordinate systems attached to the manipulator, the robot based **F**_*Robot*_ = (**x**_*R*_,**y**_*R*_,**z**_*R*_) is used as reference, the last frame **F**_*Needle*_ = (x_*N*_,y_*N*_,z_*N*_) is attached to the needle, the passive optical marker with frame **F**_*Marker*_ = (**x**_*M*_,**y**_*M*_,**z**_*M*_) is mounted on the end-effector, the other coordinate systems (x_*i*_,y_*i*_,z_*i*_), *i* = 1⋯5 are attached to the links. The transformation from marker frame to tracker base and robot frame can be expressed respectively with matrix of the form

(2) $$T_{M}^{T}=\left\{\begin{array}{cc} R_{M}^{T} & d_{M}^{T}\\ 0 & 1 \end{array}\right\},T_{M}^{R}=\left\{\begin{array}{cc} R_{M}^{R} & p_{M}^{R}\\ 0 & 1 \end{array}\right\}$$

where $p_{M}^{R}$ and $d_{M}^{T}$ are marker positions observed in **F**_*Robot *_and **F**_*Tracker*_, rotation matrix $R_{M}^{T}=\left[m_{1},m_{2},m_{3}\right]$ are axis vectors **x**_*M*_, **y**_*M*_, **z**_*M *_that expressed in tracker frame, and $R_{M}^{R}=\left[n_{1},n_{2},n_{3}\right]$ refers to the axis expression in robot frame. They are ideally related by robot-tracker transformation matrix $T_{R}^{T}$, shown as

(3) $$T_{R}^{T}=\left\{\begin{array}{cc} R_{R}^{T} & p_{R}^{T}\\ 0 & 1 \end{array}\right\}$$

(4) $$T_{M}^{T}=T_{R}^{T}T_{M}^{R}$$

Affected by measurement noise **U** and **V**_*i*_, *i* = 1, 2, 3, the expansions of (4) are

(5) $$d_{M}^{T}=R_{R}^{T}p+p_{M}^{R}+U$$

(6) $$m_{i}=R_{R}^{T}n_{i}+V_{i}$$

K corresponded pose pairs ${\left\{T_{M}^{T},T_{M}^{R}\right\}}_{k}$, *k* = 1 ⋯ *K* were recorded at different configurations of robot angle setting. Solving the optimal transformation $T_{R}^{T}$ typically requires minimizing a least square error criterion given by

(7) ∑=∑k=1K∑i=13αkimki−RRTnki2+∑k=1KβidMkT-RRTpMkR−pRT2

A dual number quaternion based algorithm was employed to estimate the transformation matrix [17], which incorporates both orientation and translation information. However, inaccuracy in robot forward kinematics seriously affects the validity of registration result. Robot calibration is required to reduce the registration error as well as inaccuracies in robot parameters of links and joint angles.

(iii). Calibration of robot parameters

The forward kinematics of the 5R1P needle manipulating robot is cascadely constructed by the transformations between consecutive joint frames based on the modified D-H parameters [18]. The needle was axially attached to the linear motor shaft, the optical marker was mounted on the outer shell of motor. The transformation matrix $T_{M}^{R}$ of maker can be read via robot forward kinematics,

(8) $$T_{M}^{R}=T_{1}^{R}T_{2}^{1}\ldots T_{5}^{4}T_{M}^{5}=F\left(X,\Theta \right)$$

(9) $$T_{i+1}^{i}=R_{x}\left(\alpha_{i}\right)T_{x}\left(a_{i-1}\right)R_{z}\left(\theta_{i}\right)T_{z}\left(d_{i}\right)R_{y}\left(\beta_{i}\right)$$

in which, **X** = (**a**, **d**, **α**, **β**, **p**)^*T*^ are link structural parameters, **a** = (*a*_1_, *a*_2_ ⋯ *a*_6_)^*T*^, **d** = (*d*_1_, *d*_2_ ⋯ *d*_6_)^*T*^, **α** = (*α*_1_, *α*_2_, ⋯, *α*_6_)^*T*^, **β** = (*β*_1_, *β*_2_, ⋯, *β*_6_)^*T*^, **p** = (*p*_*x*_, *p*_*y*_, *p*_*z*_)^*T*^ are positions of the optical marker relative to the robot end-effector, **Θ** = (*θ*_1_, *θ*_2_, ⋯ *θ*_6_)^*T *^are joint variables. Variations in robot geometric parameters due to manufacturing tolerances or component limitations account for the accuracy of robot kinematics. Considering the 2nd and 3rd joint axes are nearly parallel, only *β*_2_ is necessary. The marker pose with nominal link parameters is noted as ${\hat{T}}_{M}^{R}$, the correct pose of the marker with kinematic errors is given by $T_{M}^{R}$, it can be expressed as

(10) $$T_{M}^{R}={\hat{T}}_{M}^{R}+dT_{M}^{R}=F\left(X+\Delta X,\Theta +\Delta \Theta \right)$$

The differential translation and rotation transformation $\delta T_{M}^{R}$ can be written as

(11) $$\delta T_{M}^{R}=dT_{M}^{R}{\left({\hat{T}}_{M}^{R}\right)}^{-1}$$

Using the first-order approximation for the differential error matrix, the translation deviations **d** = [*dx*, *dy*, *dz*]^*T *^in robot frame due to parameter errors can be written in the following linear form [19]

(12) $$d=W_{\theta }\Delta \theta +W_{\alpha }\Delta \alpha +W_{a}\Delta a+W_{d}\Delta d+W_{\beta }\Delta \beta +W_{p}\Delta p$$

where *Δ***θ**, *Δ***a**, *Δ***d**, *Δ***α**, *Δ***β**, *Δ***p** refer to the disturbances in robot parameters. After N measurements of the corresponded marker positions, the identification equation is constructed as

(13) D=JΔX

in which $D={\left[d_{1}^{T},d_{2}^{T}\cdots d_{N}^{T}\right]}^{T}$, **d**_*i*_ is the *i*_*th *_measured marker position error in robot frame,

(14) $$d_{i}=p_{\mathrm{Mi}}^{R}-R_{T}^{R}\left(d_{\mathrm{Mi}}^{T}-p_{R}^{T}\right)$$

$$J={\left[W_{1}^{T}\;W_{2}^{T}\cdots W_{N}^{T}\right]}^{T}$$

is the identification Jacobian matrix, each row block **W**_*i *_refers to the *i*_*th *_coefficient matrix of **d**_*i*_, **W**_*i*_ = [**W**_*θi*_, **W**_*αi*_, **W**_*ai*_, **W**_*di*_, **W**_*βi*_, **W**_*pi*_]. The least-square estimation of robot parameter deviation *Δ***X** is calculated by the pseudo-inverse matrix **J**^† ^of **J**,

(15) $$\Delta X=J^{†}D$$

then the robot parameters can be compensated by $X=\hat{X}+\Delta X$, $\Theta =\hat{\Theta }+\Delta \Theta$. The least square method tends to change the mechanical structure of robot completely when the estimated parameters deviate a lot from the actual ones. Only 5 rotational joint zero-positions, 4 link lengths and 3 marker positions are chosen to calibrate for consistency and simplicity in solving the inverse kinematic.

(iv). Simplified two-step scheme for robot-track registration

In this section, we introduce a simplified two-step registration scheme for both robot-tracker correspondence and robot calibration. The entire registration is summarized as follow.

**Input:** corresponded frame pairs, *k* = 1 ⋯ *K* of the optical makers measured via optical tracker and robot forward kinematics respectively;

**Output:** transformation $T_{R}^{T}$ and robot parameters (**X**, **Θ**);

**Initialization:** robot parameters (**X**_0_, **Θ**_0_) are initialized by the nominal settings;

**Iteration:** for n = 1 to *n*_max _or the registration error *Σ* converges, do

1. Compute the transformation matrix $T_{R}^{T}$ by minimizing the object function (7);

2. Update the marker positions and deviation matrix using the newer robot kinematics *F*(**X**_*n*_, **Θ**_*n*_);

3. Calibrate and compensate robot parameters

(16) $$\begin{array}{c} X_{n}=X_{n-1}+\Delta X\\ \Theta_{n}=\Theta_{n-1}+\Delta \Theta \end{array}$$

4. End the iteration when n = *n*_max_ or the decrease of the MSE below a threshold h.

### Control scheme

With the image-guidance interface, the surgeon telemanipulated the robot to approach the insertion point manually in free space, needle alignment and interventional puncture are performed autonomously in accordance with the surgical planning. In this study, the haptic device acted as the master controller, while the 5R1P robot performed as the slave needle operator. The master and the slave were connected through a communication network.

(i). Master–slave control for manually needle approaching

Since the operation space of the master is not in proportion to that of the slave, a joint-joint velocity scaling control was applied to the master–slave system. Operations on the master side were scaled down to the slave side directly, the master joint velocities ${\dot{\Theta }}_{\text{master}}$ were mapped to the corresponding slave joint velocities ${\dot{\Theta }}_{\text{slave}}$ by

(17) $$\begin{array}{ll} {\dot{\Theta }}_{\text{slave}}=\Lambda {\dot{\Theta }}_{\text{master}} & \;\Lambda =\mathrm{diag}\left(\lambda_{1},\lambda_{2},\cdots \lambda_{6}\right) \end{array}$$

where **Λ** is a scaling diagonal matrix, different scaling ratio was assigned to each joint pair according to their contributions to the translation and rotation of the end-effector. Small ratio helps reduce disturbances in manual input. The calculated joint velocities were then sent to the Mitsubishi alternate current servo-unit, all five joints were controlled simultaneously to approach the puncture point, the linear motor were controlled by safe button on the joystick of master separately for needle insertion.

(ii). Optical tracker feedback control for needle alignment

Inaccuracy of robot-tracker correspondence and robot parameters impacts the absolute precision severely when using the internal control system of the robot itself. But since the relative accuracy is better than the allowed tolerances the robot can be adjusted until the absolute accuracy is good enough [20,21]. This section presents an optical tracker feedback control method to improve the accuracy of needle alignment for manual or robotic needle steering operations in soft tissue.

Once the needle tip approached the puncture point, autonomous needle alignment is conducted in accordance with the surgical planning $T_{\mathrm{EdR}}^{T}$ in tracker frame. In needle alignment stage, the needle shaft maintains straight and without touching the tissue, the needle tip pose measured by optical tracker is noted as $T_{\mathrm{EdC}}^{T}$ in tracker frame and $T_{\mathrm{EdC}}^{R}$ in robot frame. The robot reports nominal needle pose $T_{\mathrm{EdN}}^{R}$, which does not ensure accuracy due to disturbances in robot parameters *Δ***X** and *Δ***Θ**. Inaccuracies also appear in the measured pose $T_{\mathrm{EdC}}^{R}$ due to robot-tracker transformation error $\Delta T_{R}^{T}$. Deviations between the measured $T_{\mathrm{EdC}}^{T}$ and the reference $T_{\mathrm{EdR}}^{T}$ are noted as $T_{E}^{T}$ in tracker frame and $T_{E}^{R}$ robot frame.

$$T_{\mathrm{EdN}}^{R}=F\left(\hat{X},\hat{\Theta }\right)$$

(18) $$T_{\mathrm{EdC}}^{R}=F\left(\hat{X}+\Delta X,\hat{\Theta }+\Delta \Theta \right)={\left(T_{R}^{T}\right)}^{-1}T_{\mathrm{EdC}}^{T}$$

(19) $$T_{E}^{T}=T_{\mathrm{EdR}}^{T}{\left(T_{\mathrm{EdC}}^{T}\right)}^{-1}$$

(20) $$T_{E}^{R}={\left(T_{R}^{T}\right)}^{-1}T_{E}^{T}$$

(21) $$T_{E}^{R}=T_{\mathrm{EdN}}^{R}{\left(T_{\mathrm{EdC}}^{R}\right)}^{-1}={\left(T_{R}^{T}\right)}^{-1}T_{E}^{T}T_{R}^{T}$$

(22) $$T_{R}^{T}={\hat{T}}_{R}^{T}+\Delta T_{R}^{T}$$

**Figure 4:**
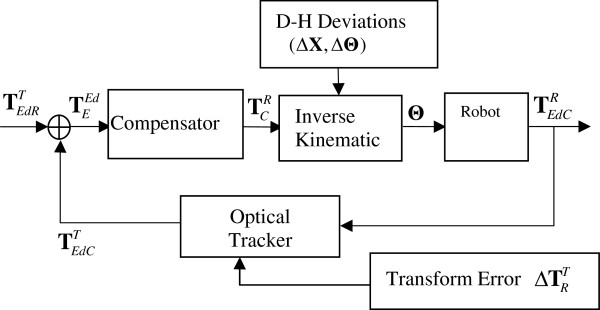
Optical tracker feedback control for needle alignment.

The goal is to make $T_{\mathrm{EdC}}^{T}$ to be close to $T_{\mathrm{EdR}}^{T}$ as possible while robust to inaccuracy in robot-tracker calibration. $\hat{X}$, $\hat{\Theta }$ are estimated robot parameters, ${\hat{T}}_{R}^{T}$ is estimated the robot-tracker transformation matrix, *Δ***X** and *Δ***Θ**, $\Delta T_{R}^{T}$ are deviations. The goal is achieved by commanding the robot to a new pose iteratively by error compensation. The control scheme is shown in Figure 3. Here we outline the optical tracker feedback control scheme as follow (Figure 4).

For k =0 to *k*_*max*_, do

1. Initialize the pose of end-effector $T_{C0}^{R}={\left({\hat{T}}_{R}^{T}\right)}^{-1}T_{\mathrm{EdR}}^{T}$ by the estimated ${\hat{T}}_{R}^{T}$;

2. Solve the inverse kinematics of robot $\Theta_{k}=F^{-1}\left(\hat{X},\hat{\Theta },T_{\mathrm{Ck}}^{R}\right)$, command the joints move to **Θ**_*k*_;

3. Measure the actual pose of end-effector $T_{\mathrm{EdC}}^{R}={\left({\hat{T}}_{R}^{T}\right)}^{-1}T_{\mathrm{EdC}}^{T}$ using optical tracker, and compute the error,

$$T_{E}^{R}={\left(T_{\mathrm{EdC}}^{R}\right)}^{-1}T_{\mathrm{EdR}}^{R}={\left(T_{\mathrm{EdC}}^{T}\right)}^{-1}T_{\mathrm{EdR}}^{T}$$

4. Modify a new command by error compensation $T_{\mathrm{Ck}+1}^{R}=T_{\mathrm{Ck}}^{R}T_{E}^{\mathrm{Ed}}$, go to step 2;

5. Stop until iteration time *k* = *k*_max_ or the error below threshold h.

## Results and discussion

Three experiments were conducted to validate the efficiency of robot-tracker registration method and the optical tracker feedback control for needle alignment task.

### Robot-tracker calibration

The correspondence of robot-tracker as well as the robot parameters were calculated by the simplified two-step scheme proposed in section II. Nominal link parameters were listed in Table 1. Corresponded frame pairs $\left\{T_{M}^{R},T_{M}^{T}\right\}$ of the maker that fixed on the end-effector were collected via optical tracker and robot nominal forward kinematics at 72 different configurations of joint settings (degree) *θ*_1_ = 20*i*, *i* = − 1, 0, 1; *θ*_2_ = − 90 + 20*j*, *j* = 0, 1; *θ*_3_ = 20*k*, *k* = 0, 1; *θ*_4_ = 15*l*, *l* = − 1, 0, 1; *θ*_5_ = 45 + 15*m*, *m* = 0, 1. A geometrical robot-tracker calibration was conducted for comparison. The end-effector moved along semicircle paths by driving joint 1 and 2 individually, the orthogonal joint axes were calculated by circle fitting to estimate the robot base. Robot parameter calibration was carried out by the least square method using the corresponded pairs $\left\{T_{M}^{R},T_{M}^{T}\right\}$. In this method, robot- tracker registration and robot calibration were conducted in sequence, additional data were required. Only 5 joint zero-positions, 4 link lengths and 3 positional parameters of the marker were chosen to calibrate. The calibrated robot parameters are listed in Table 2, there wasn’t obvious difference between these methods.

**Table 1:** The nominal parameters of robot

**Joint**	**a(mm)**	**α(rad)**	**d(mm)**	**θ(rad)**
1	0.00	0.00	0.00	0.00
2	100.00	−1.57	0.00	0.00
3	290.00	0.00	0.00	0.00
4	121.00	−1.57	310.00	0.00
5	0.00	1.57	0.00	0.00
6	0.00	−1.57	0.00	0.00
Position of maker (mm): (40.00, 0.00, 160.00)				

**Table 2:** Calibrated robot parameter

**Joint**	**a(mm)**	**α(rad)**	**d(mm)**	**θ(rad)**
1	0.00	0.00	0.00	0.0059
2	99.23	−1.57	0.00	−0.0179
3	291.77	0.00	0.00	0.0119
4	119.62	−1.57	310.00	0.0024
5	0.00	1.57	0.00	−0.0138
6	0.00	−1.57	−0.00	0.00
Position of maker (mm): (40.32, 0.71, 158.02)				

**Figure 5:**
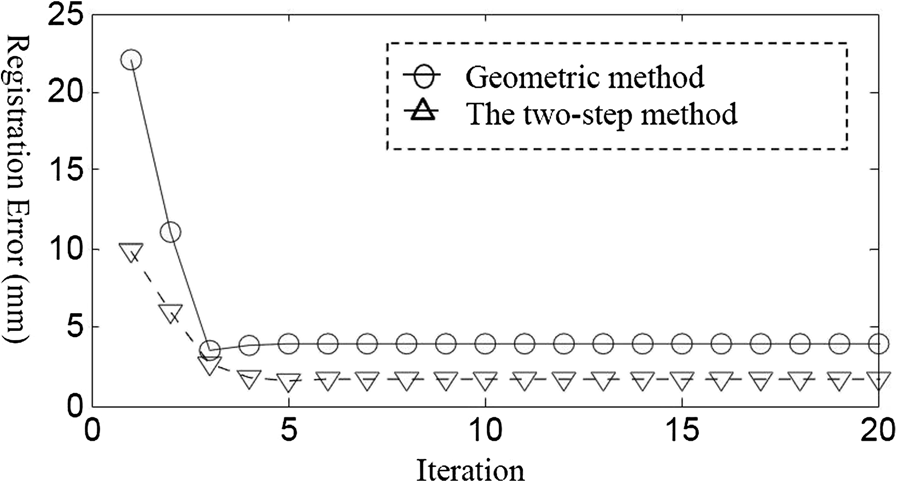
Registration error by two-step method and geometric method.

A fixed robot-tracker correspondence was used in the geometric method, while iterative searching for the optimal $T_{R}^{T}$ was employed in the simplified scheme, the rotational and translational differences between the estimated matrices $T_{R}^{T}$ were (0.0003,0.0016,0.0008) rad and (0.3399, -0.1184, -0.9176)mm. As shown in Figure 5, the registrated MSE errors of marker position were plotted, the simplified method performed better than the geometric method both in accuracy and speed. Residual error still remained after the registration procedure due to the linearization of error model and the inherited positioning error of the optical tracker. Open-loop control can’t eliminate these residual error, it is necessary to design a closed-loop scheme to compensate the influence caused by robot parameter deviations.

### Optical tracker based error compensation experiment

This experiment is to evaluate how the errors in robot-tracker correspondence and robot parameters affect the needle alignment precision and how they are compensated with the optical tracker feedback control. Gaussian distributed *N*(0, *σ*^2^) disturbances are introduced to link lengths, joints zero-position, position of the optical marker and robot-tracker transformation matrix $T_{R}^{T}$, the angular disturbance in joint angles and orientation of $\Delta T_{R}^{T}$ ranges over (0 ~ 0.4) radians, while the length disturbance in robot links, marker position and translation part of $\Delta T_{R}^{T}$ varies from 0 to 100 mm. Their influence on the precision of needle alignment were analyzed both independently and jointly. In this experiment, the robot was commanded to a fixed pose $T_{\mathrm{EdR}}^{T}$, the positional errors of needle tip and rotational errors of needle shaft were measured by optical tracker after the open loop positioning. The translational error δ*p* refers to the deviation of needle tip **P**_*N*_ to the target **P**_*R*_, rotational error is the difference δ*v* between the actual orientation **v**_*N*_ of needle shaft and the referenced direction **v**_*R*_,

$$\mathrm{\delta p}=∥p_{N}-p_{R}∥$$

$$\mathrm{\delta v}=\arccos\left(v_{N},v_{R}\right)\approx ∥v_{N}-v_{R}∥$$

the approximation holds only for small directional deviations. To compensate robot parameter disturbances, the robot was driven to the modified poses iteratively, and the minimum error was selected in 10 iterations with position threshold 0.2. In this case, the same target pose was used in both stages.

**Figure 6:**
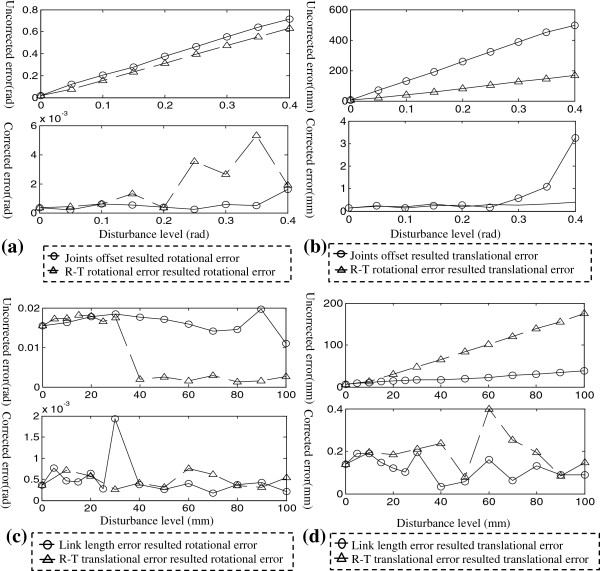
Positioning error of the needle shaft measured by tracker before and after feedback compensation.

Figure 6(a-b) illustrate the influences of joint disturbances and rotational error of $T_{R}^{T}$ individually, the final pose of the needle shaft goes far away from the reference dramatically as the disturbance level grows, the feedback scheme can limit these errors in a reasonable range. As shown in Figure 6(c-d), the positioning error grows linearly with disturbances in link lengths and translation part of $T_{R}^{T}$, the feedback control performs stable over these variations.

The influence of disturbance in robot parameters was also studied jointly. Table 3 outlines nine levels of combined disturbance in robot parameters. Table 4 lists the results of the feedback control for these cases. The results indicate that even with significant 10 centimeters error in link lengths and robot-tracker translational part, 0.45 radian in joint offsets and optical robot-tracker rotational part, 1 mm positioning accuracy and highly rotational precision can be easily achieved. Even though the feedback control is capable of compensating large range disturbances, the iteration times and correction step grows linearly with the disturbance level, a calibration process is necessary to reduce the initial error and limits the movement magnitude.

**Table 3:** Combined disturbance levels in robot parameters

**Set name**	**Link length error (mm)**	**Joint angle error (rad)**	**Marker position (mm)**	**Robot-tracker orientation (rad)**	**Robot-tracker displacement (mm)**
No. 1	1.00	0.05	1.00	0.05	1.00
No. 2	3.00	0.10	3.00	0.10	3.00
No. 3	5.00	0.15	5.00	0.15	5.00
No. 4	10.00	0.20	10.00	0.20	10.00
No. 5	20.00	0.25	20.00	0.25	20.00
No. 6	30.00	0.30	30.00	0.30	30.00
No. 7	40.00	0.35	40.00	0.35	40.00
No. 8	50.00	0.40	50.00	0.40	50.00
No. 9	60.00	0.45	60.00	0.45	60.00

**Table 4:** Error magnitudes of optical tracker feedback control

**Set name**	**Uncorrected translational error (mm)**	**Uncorrected rotational error (rad)**	**Corrected translational error (mm)**	**Corrected rotational error (E-4 rad)**	**Iteration times**
No. 1	67.078	0.08	0.25	5.98	6
No. 2	123.18	0.15	0.10	4.60	7
No. 3	175.65	0.21	0.16	8.78	9
No. 4	223.32	0.27	0.35	6.20	10
No. 5	263.04	0.31	0.26	3.75	15
No. 6	299.30	0.35	0.26	2.06	20
No. 7	334.79	0.39	0.29	4.42	30
No. 8	357.12	0.43	0.30	5.16	39
No. 9	380.93	0.43	0.55	7.72	50

### Robot assisted needle insertion experiment

A triple-modality (CT, MR, US) abdominal phantom model 057 from Computerized Imaging Reference Systems (CIRS) was used for the in vivo data Test. The internal structure of the model 057 includes partial abdominal aorta, partial vena cava, spine and two partial kidneys each with a lesion. The lesions are high contrast relative to the background in MR and can be barely identified in US.

First, the phantom was scanned with Siemens MAGNETOM Trio Tim 3.0 T machine, meanwhile the robot was calibrated to the optical tracker frame following the process in section 2. To avoid the accumulated US-MR registration error in robot assisted needle insertion experiment, 7 silicon square makers were attached to the surface of phantom, a rigid registration was employed to transform the MR image to optical tracker frame directly by the corresponded position pairs of the silicon markers both in MR image frame and optical tracker frame.

**Figure 7:**
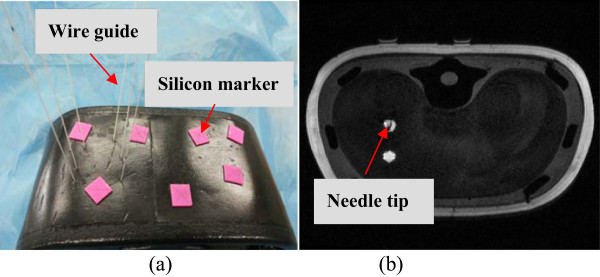
Results of robot assisted needle insertion on kidney phantom.

And then, six planned trajectories were defined, each including an entry point on the skin and a target point within a lesion near the left kidney. All trajectories were transferred into the optical tracker frame, the robot was commanded to complete the needle alignment operation autonomously using the optical tracker feedback control. Once the needle shaft was aligned along the planed direction outside the phantom, the linear motor was controlled to drive the needle to the desired depth by safe button on the joystick of master, a NiTi alloy wire guide(RFSPC-035145-0-I-AQ, Cook Urological Incorporated) was inserted into the target lesion through the trocar to trace the insertion trajectory afterwards(seen in Figure 7(a)). After all six insertions were finished, the phantom was scanned with the Siemens MAGNETOM Trio Tim 3.0 T machine again to evaluate the final insertion accuracy, the needle-target distance was measured based on the multiplanar reconstructed images (seen in Figure 7(b)).

The position and orientation of needle shaft were also measured after needle alignment by the optical tracker, Table 5 lists the results of robot assisted needle insertion experiment. The needle-target distance over the six insertion trails was 2.15 ± 0.17 mm, difference between the six tests was relatively small, indicating a repeatable performance for the six different insertion trajectories. The total needle insertion error come from the image-tracker registration error 1.13 ± 0.31 mm, optical tracker positioning error 0.18 ± 0.14 mm for passive rigid markers [22], robot assisted needle alignment error 0.24 ± 0.08 mm, needle deflection and phantom deformation.

**Table 5:** Results of robot assisted needle insertion experiment on kidney

**PATHs**	**1**	**2**	**3**	**4**	**5**	**6**	**Mean**	**Std. Dev.**
Position error of alignment (mm)	0.14	0.29	0.14	0.30	0.23	0.32	0.24	0.08
Direction error of alignment (E-4 rad)	4.88	6.08	6.28	6.00	8.01	9.44	6.78	1.65
Position error of insertion (mm)	2.35	2.10	2.34	2.10	1.89	2.11	2.15	0.17
Operation time(s)	84	79	81	77	75	80	79.33	3.14

In previous study [3], the accuracy of the volume navigation was evaluated via puncture tests on a customized phantom. The mean needle-target distance was 2.7 mm for the trials performed by an experienced radiologist, while 3.1 mm for a medical resident without experience. With the help of the optical tracker based feedback control, precise needle alignment could facilitate the follow-up manual needle insertion or robotic needle steering. When the positioning accuracy of tracking system increases, the absolute positioning accuracy of needle alignment will increase. However, in needle steering stage, the positioning information from the tracker was incorrect due to bending of needle shaft in soft tissue. Further work will use magnetic sensor to track the precise needle tip or intra-operation visual servoing technique, more dexterous needle steering inside tissue will be studied.

## Conclusions

This paper presents an integrated needle operation robot system for percutaneous renal intervention. A simplified image-tracker-robot registration procedure was introduced. Variations in robot geometric parameters and tracker-robot correspondence account for the needle positioning accuracy of robot. An optical tracker feedback control was proposed and validated to compensate these disturbance for needle alignment. The accuracy is inherited from the optical positioning system. Experiments show that the control scheme is capable of providing accurate 3D needle alignment, and compensating wide range of disturbance in robot parameters and tracker-robot correspondence. Robot assisted needle insertion experiments were performed on kidney phantom, precise needle alignment could improve the precision of needle insertion. Robot-assisted needle steering has the potential to improve the accuracy through more dexterous control of the needle-tip trajectory, further work will involve tip/base needle manipulation for needle steering in soft tissue [23].

## Competing interests

The authors declare that they have no competing interests.

## Authors’ contributions

DWZ implemented the robot teleoperation framework, robot-tracker registration and error compensation algorithm. ZCL and KC were responsible image guided framework, 3D reconstruction, MR to US registration. XPZ participated in the robot servoing algorithm design. LW provided the experiment infrastructure and contributed to the result discussion. All authors read and approved the final manuscript.
